# Alterations of the default mode network, salience network, and frontoparietal network in non-problematic Internet use and their association with mood regulation: from an internet literacy perspective

**DOI:** 10.3389/fnins.2025.1599987

**Published:** 2025-07-01

**Authors:** Mami Shibata, Kosuke Tsurumi, Kei Kobayashi, Sayaka Yoshimura, Michael Spantios, Naoya Oishi, Toshiya Murai, Hironobu Fujiwara

**Affiliations:** ^1^Department of Psychiatry, Graduate School of Medicine, Kyoto University, Kyoto, Japan; ^2^Department of Neurodevelopmental Psychiatry, Habilitation and Rehabilitation, Graduate School of Medicine, Kyoto University, Kyoto, Japan; ^3^Organization for Promotion of Neurodevelopmental Disorder Research, Kyoto, Japan; ^4^Department of Developmental Disorders, National Institute of Mental Health, National Center of Neurology and Psychiatry, Tokyo, Japan; ^5^Human Brain Research Center, Kyoto University Graduate School of Medicine, Kyoto, Japan; ^6^RIKEN Center for Advanced Intelligence Project Decentralized Big Data Team, Tokyo, Japan; ^7^The General Research Division, Osaka University Research Center on Ethical, Legal and Social Issues, Osaka, Japan

**Keywords:** non-problematic internet use, magnetic resonance imaging, mood regulation, functional connectivity, triple-network model, behavioral addiction

## Abstract

**Introduction:**

Although a number of neuroimaging studies on problematic internet use (PIU) have been conducted, few studies on non-problematic internet use (non-PIU) are available; therefore, the influences of non-PIU on brain function are unclear. Resting-state functional connectivity (rsFC) could be an appropriate tool to investigate subclinical samples, such as non-PIU, to capture the potentially subtle changes compared to clinical samples. In the context of rsFC, the default mode network (DMN), the central executive network (CEN), and the salience network (SN) are representative of the brain networks. Because accumulating studies have investigated rsFC between these networks in PIU samples, investigating rsFC between these networks in non-PIU samples could give us a clue to elucidate the neural basis of non-PIU in relation to those of PIU.

**Methods:**

We recruited 119 healthy volunteers and used the General Problematic Internet Use Scale-2 (GPIUS-2) to measure their propensity to use the Internet. The GPIUS-2 consists of four subscales. We analyzed the correlation between the degree of Internet use assessed by the GPIUS-2 and the rsFC of the regions comprising DMN, CEN, and SN using resting-state functional magnetic resonance imaging in non-PIU samples.

**Results:**

There was a positive correlation between the mood regulation subscale score and SN (bilateral anterior insula) – CEN (left lateral prefrontal cortex: LPFC), SN (left anterior insula) – DMN (medial prefrontal cortex: MPFC), and DMN (MPFC) – CEN (right LPFC) rsFC. Additionally, there was a negative correlation between the mood regulation subscale score and DMN – FPN rsFC.

**Discussion:**

The correlations between rsFC and non-PIU suggest that non-PIU and PIU could influence rsFC in opposite directions. Furthermore, the hub regions comprising connections correlated with non-PIU in this study are involved in cognitive and emotional processes. One possible interpretation would be that the bilateral insula and synchronized LPFC activity in individuals with non-PIU results in well-functioning emotion regulation as an appropriate coping strategy, in contrast to PIU. These findings suggest that non-PIU might contribute to health promotion through mood regulation with alterations in the functional connectivity between the regions involved in mood regulation.

## 1 Introduction

The Internet has been established as a necessary tool for addressing many social issues. However, its risks include behavioral addiction (Billieux et al., 2015; Müller et al., 2022; Pontes, 2022), with problematic Internet use (PIU) being a notable concern. PIU encompasses gaming disorders, problematic pornography, social network addiction, and compulsive browsing (Pawlikowski et al., 2014; Pontes, 2022; Rendi et al., 2007). One meta-analysis reported that 85% of magnetic resonance imaging (MRI) studies on PIU were conducted in patients with gaming disorders (Sepede et al., 2016). In these studies, PIU was associated with poor performance in attention-demanding tasks (Park et al., 2011), impaired decision-making skills (Sun et al., 2009), and daily cognitive failures (Hadlington, 2015).

Research using structural and functional imaging is accumulating knowledge on the neural basis of PIU. Structural neuroimaging studies have revealed alterations related to PIU, such as volume reduction in the prefrontal cortex (PFC) (Jin et al., 2016; Wang et al., 2018; Yuan et al., 2011). In functional neuroimaging studies, PIU participants showed altered brain activity in a variety of brain regions, including the insula, anterior cingulate cortex, and caudate during a monetary task (Dong et al., 2013); the inferior parietal lobule and PFC during an Addiction Stroop Task (Zhang et al., 2016); and the sensorimotor area during a Go/No-go task (Chen et al., 2015).

Recently, functional connectivity (FC), defined as the temporal correlation between neural activity patterns in different brain regions (Aertsen et al., 1989; Friston, 1994), has been investigated. Human brain activity is topologically organized into a set of spatiotemporal networks with phase relationships, often referred to as large-scale brain networks, which orchestrate disparate cognitive processes. The “triple-network model” hypothesizes that three core intrinsic neurocognitive networks—the frontoparietal network (FPN), the default mode network (DMN), and the salience network (SN)—interact with each other within the framework of large-scale networks (Menon, 2011). The FPN and SN typically show increased activation during cognitive tasks. In contrast, the DMN is deactivated during cognitively demanding tasks, and activated during rest (Kamp et al., 2018). The SN plays a pivotal role in switching between the FPN and DMN by allocating attention, resulting in a negative correlation between the FPN and DMN activity (Fox et al., 2005). In the triple-network model, an increase in functional connectivity between the SN and DMN has been observed in patients with depression, which is often comorbid with PIU (Jiang et al., 2017; Posner et al., 2017; Stern et al., 2012). Several studies have revealed increased SN-DMN connectivity in patients with PIU (Hong et al., 2018; Zhang et al., 2017), although a few have reported decreased SN-DMN connectivity (Wang et al., 2017). One study has reported decreased DMN-FPN connectivity in patients with PIU (Siste et al., 2022).

From the perspective of therapeutic intervention for PIU, unlike other addictions, abstaining from internet use is not always realistic because of its social importance. Moreover, non-PIU could benefit mental health and cognitive abilities (Fujiwara et al., 2018; Huang et al., 2021; US Food and Drug Administration, 2020). Furthermore, adequate Internet literacy has been reported to be associated with self-efficacy in Internet use, including exchanging e-mails or browsing (Huang et al., 2021). Notably, the Internet, presumably under adequate regulation by users, could be applied for cognitive control training for instituting preventive intervention for depression (Hoorelbeke and Koster, 2017) and improving attention and motivation in neurocognitive disorders (Miller et al., 2013; Robert et al., 2020). Game-based devices have been approved by the U.S. Food and Drug Administration as interventions for symptoms of attention deficit hyperactivity disorder (2020).

However, the neural basis of non-PIU remains unclear because neuroimaging studies of non-PIU are scarce. In one of the few reports where moderate use of the Internet could have a positive effect on neural connectivity, video game experts showed higher FC between the SN and FPN than amateur players, presumably resulting from trained attention and working memory, which are needed to play video games (Gong et al., 2016). In another study, the tendency for Internet use was positively correlated with FC in the resting state in reward/motivation networks, indicating that subclinical levels of the Internet use might act to maintain reward/motivation-network integrity (Fujiwara et al., 2018).

It is possible that the degree of change in neural connectivity in non-PIU groups is not as drastic as that in PIU groups, which may be why there are very few reports on non-PIU. Resting-state FC could be an appropriate tool for studying non-PIU samples to capture potentially subtle changes compared to PIU samples. The purpose of this study is to investigate the relationship between Internet use and resting-state FC within the framework of the triple-network model in healthy participants, and compare it with previously reported alterations of the triple-network in PIU. We hypothesized that resting-state FC between the SN and FPN would be increased in the non-PIU group compared to the PIU group, based on studies showing positive effects on mental state.

## 2 Materials and methods

### 2.1 Participants

In the current study, we set the effect size to 0.3, referring to previous studies (Schmitgen et al., 2022; Mizumoto and Takeuchi, 2011). If we set the effect size to moderate (*r* = 0.30), the significance level to 5%, and the power to test to 80%, the sample size would be 84 participants. However, in this study, it was necessary to take into account the possibility of incomplete data collection and dropout cases, and we considered it appropriate to set the sample size at 100 cases. Furthermore, we referred to a previous, similar study that examined the association between the Internet and FC (Vergara et al., 2022). Given these considerations, we targeted a sample size of about 100 individuals. We recruited 119 healthy right-handed volunteers (73 [62.9%] men) from May 2017 to January 2019 with a mean age of 36.20 (standard distribution (*SD*] = 14.31) years. We included native speakers of Japanese aged 18–70 years at the time of registration. The Structured Interview for Diagnostic and Statistical Manual of Mental Disorders (SCID; American Psychiatric Association [APA], 2013) was performed by well-trained psychiatrists to exclude psychiatric and neurological disorders and diseases such as mood, anxiety, and alcohol use disorders. The concurrent judgment of two well-trained psychiatrists was used to determine that none of the participants had any psychiatric or neurological illnesses. The Japanese version of the Adult Reading Test (Matsuoka et al., 2002) was used to confirm that all participants fell within the normal range of intelligence. After the experimental procedures were fully explained, all participants provided written informed consent before study participation. The Ethics Committee of the Kyoto University Graduate School and Faculty of Medicine reviewed and approved this study (R0879, January 17th, 2017), which was conducted in accordance with the Declaration of Helsinki. We did not have access to information that could identify the individual participants during or after data collection.

### 2.2 Psychological questionnaire

The General Problematic Internet Use Scale-2 (GPIUS-2) was used to measure PUI. The GPIUS-2 is a self-rating questionnaire containing 15 items, responses are scored on an 8-point Likert scale (1, strongly disagree; 8, strongly agree). The GPIUS-2 scores ranged from 15 to 120. Higher GPIUS-2 scores indicate a more severe tendency toward Internet addiction. Thus, the original GPIUS-2 was validated. The internal consistency reliability of the GPIUS-2 (Cronbach’s alpha, 0.78–0.85) indicated a high level of reliability (Caplan, 2002). GPIUS-2 is a multidimensional scale composed of four subscales: preference for online social interaction (POSI), use of the Internet for mood regulation (MOOD), deficient self-regulation (SELF, further divided into cognitive preoccupation and compulsive Internet use), and negative outcomes (NEGA).

The POSI assesses the preference for Internet-based communication over face-to-face interaction. The MOOD assesses the tendency to use the Internet to regulate unpleasant emotional states (e.g., “I have used the Internet to make myself feel better when I’ve felt upset/lonely/depressed”).

Deficient self-regulation indicates a tendency toward a decreased self-monitoring state in which conscious self-control is consciously diminished because of mechanisms such as a growing habit of using the Internet. Deficient self-regulation, in some cases, can be categorized into “cognitive preoccupation” (e.g., “When I haven’t been online for some time, I become preoccupied with the thought of going online”) and “compulsive internet use” (e.g., “I find it difficult to control my Internet use”). Furthermore, deficient self-regulation assesses difficulty in appropriately monitoring and judging an individual’s Internet usage patterns and adjusting Internet-related behaviors (Caplan, 2010). Negative outcomes evaluated the adverse consequences of excessive Internet use. We used the Japanese version of the GPIUS-2, the reliability and validity of which have been previously confirmed (Yoshimura et al., 2022). The internal consistency reliability of the Japanese version of the GPIUS-2 total scores and its subscales (Cronbach’s alpha, 0.75–0.79) showed high reliability. The correlation coefficient between the Japanese version of the GPIUS-2 and the Internet Addiction Test was 0.75 (*p* < 0.001), indicating satisfactory validity (Yoshimura et al., 2022).

The Beck Depression Inventory-II (BDI-II) assesses the severity of depressive symptoms (Beck et al., 1996). This scale comprises 21 items with scores ranging from 0 to 63. Higher BDI-II scores indicate more severe depressive tendencies. We used the Japanese version of the BDI-II, the validity of which has been previously confirmed by the Center for Epidemiologic Studies Depression Scale. Internal consistency reliability (Cronbach’s alpha, 0.87) and item homogeneity (mean inter-item correlation coefficient, 0.24) were also confirmed (Griffanti et al., 2014). We used the BDI-? as a screening tool for depression at the clinical level.

### 2.3 MRI acquisition

MRI acquisition was performed with a 40-mT/m gradient and a receiver-only 32-channel phased-array head coil on a 3-Tesla MRI unit (Tim-Trio; Siemens, Erlangen, Germany). A rsfMRI was performed. A 360-s rsfMRI scan was obtained using a single-shot gradient-echo planar imaging (EPI) pulse sequence. The imaging time of rsfMRI was determined based on previous studies in the field of behavioral addiction (Tsurumi et al., 2020; Bellmunt-Gil et al., 2023; Wang et al., 2022; Bordier et al., 2022). The participants were instructed to keep looking at the cross displayed on the monitor without thinking about anything specific during the resting-state condition. Structural MRI data were acquired using three-dimensional magnetization-prepared rapid gradient-echo sequences. The parameters for resting-state data were as follows: echo time, 30 ms; repetition time, 2,500 ms; flip angle, 80°; field of view, 212 × 212 mm; matrix size, 64 × 64; in-plane spatial resolution, 3.3,125 × 3.3,125 mm^1^ ; 40 total axial slices; and slice thickness, 3.2 mm with 0.8 mm gaps in ascending order. The parameters for the three-dimensional magnetization-prepared rapid gradient-echo images were as follows: echo time, 3.4 ms; repetition time, 2,000 ms; inversion time, 990 ms; field of view, 225 × 240 mm; matrix size, 240 × 256; resolution, 0.9,375 × 0.9,375 × 1.0 mm^2^ ; and 208 total axial sections without intersection gaps. A dual-echo gradient-echo dataset for B0-field mapping was acquired for distortion correction. Head movement was minimized within the head coil using a foam rubber pad.

### 2.4 Image pre-processing

Using field map data, the rsfMRI dataset was corrected for EPI distortion via the Functional Magnetic Resonance Imaging of the Brain (FMRIB) Utility for Geometrically Unwarping EPIs, which is part of the FMRIB Software Library software package (ver. 5.0.9;^3^ Fmrib Analysis Group, 2015, Oxford, United Kingdom). Moreover, artifact components and motion-related fluctuations were removed from the images using FMRIB’s Independent Component Analysis (ICA)-based Xnoiseifier (Griffanti et al., 2014). After pre-processing, we processed the structural and functional MRI data using the functional connectivity toolbox (CONN) (ver. 17e;^4^ Gabrieli Laboratory, Cambridge, MA, United States) and Statistical Parametric Mapping software package (ver. 12;^5^ Wellcome Trust Center for Neuroimaging, 2012, London, United Kingdom) (Whitfield-Gabrieli and Nieto-Castanon, 2012).

The following pre-processing steps were conducted using a default pre-processing pipeline for volume-based analysis in the Montreal Neurological Institute space (Montreal, Canada). All functional images were pre-processed, including realignment, unwarping, slice-timing-corrected, co-registration with structural data, spatial normalization to the standard Montreal Neurological Institute space, outlier detection [Artifact Detection Tools-based scrubbing (ART)],^6^ and smoothing with a Gaussian kernel with a full width at half maximum of 8 mm. Structural data were segmented into gray matter, white matter (WM), and cerebrospinal fluid (CSF) and normalized using the same default pre-processing pipeline. Principal components of signals from the WM and CSF, as well as translational and rotational movement parameters (with another six parameters representing their first-order temporal derivatives), were removed using covariate regression analysis by CONN. Using the implemented CompCor strategy, the effects of nuisance covariates, including fluctuations in fMRI signals from the WM, CSF, and their derivatives, as well as the realignment parameter noise, were reduced. As recommended in a previous study, we performed bandpass filtering with a frequency window of 0.01–0.1 Hz (Sun et al., 2009). We observed that this pre-processing step increased retest reliability. Before running the FMRIB’s ICA-based Xnoiseifier, we used frame-wise displacement to evaluate head movement during fMRI, which quantifies the head motion between each volume of functional data. Participants were excluded if the number of volumes in which the head position was 0.5 mm different from the adjacent volumes was more than 15% (Power et al., 2012).

### 2.5 FC analysis

#### 2.5.1 ROI-to-ROI FC analyses

The region of interest (ROI)-to-ROI FC was calculated using the CONN toolbox (Gabrieli Laboratory, 2017). We adopted the ROIs defined from CONN’s ICA of the Human Connectome Project dataset. The ROIs were grouped into several networks, diameters, and peak coordinate bases.

The SN comprises seven areas: the anterior cingulate cortex (0, 22, 35), left and right anterior insula (left: –44, 13, 1; right: 47, 14, 0), left and right rostral PFC (left: −32, 45, 27; right: 32, 46, 27), and the bilateral supramarginal gyrus (left: −60, −39, 31; right: 62, −35, 32). The DMN comprised four areas: the medial PFC (MPFC) (1, 55, −3), left and right lateral parietal lobes (left: −39, −77, 33; right: 47, −67, 29), and the posterior cingulate cortex (1, −61, 38). The FPN comprised four areas: the left and right lateral PFC (LPFC) (left: –43, 33, 28; right: 41, 38, 30), and the left and right posterior parietal cortices (left: −46, −58, 49; right: 52, −52, 45). The preprocessed fMRI time series of all voxels in the 15 ROIs were extracted and averaged for each participant. ROI-to-ROI FC was defined as the Fisher-transformed bivariate correlation coefficient for each pair of 15 regions (Z_*i, j*_: Z score between the ith and jth ROI), which resulted in a 15 × 15 correlation matrix (105 FCs) for each participant.

#### 2.5.2 inter-network FC analyses

For each of the three networks, the inter-network FC strength was defined as the mean FC of all of the possible connections, i.e., *Z*_*X*,*Y*_ = 1/*n*_*X*_*n*_*y*__∑*i* ∈ *X*,*j* ∈ *Y*_|*Z*_*i*,*j*_| where n_*x*_ is the number of ROIs within a specific network X. X and Y represent one and another network of the three networks (Wang et al., 2015).

### 2.6 Statistical analyses

Kolmogorov–Smirnov tests were performed to test whether age and total and subscale GPIUS-2 scores were normally distributed. Additionally, to assess the differences in age and scores by sex, the student’s *t*-test (two-tailed) was applied for items with a normal distribution, while the Mann–Whitney U test was used unless the items were normally distributed. Correlation analyses among the items were performed using Pearson’s correlation coefficient or Spearman’s rank correlation coefficient depending on the normality of the data distribution. These tests were performed using SPSS Version 22.0.0 (International Business Machines Corporation, 2013).

#### 2.6.1 ROI-to-ROI FC analyses

We assessed the associations between the GPIUS-2 total score and its subscales and the FC values between each ROI. The associations between the GPIUS-2 scores and FC values of the two ROIs were calculated using t-statistics by CONN, with age, sex, and BDI-II scores as covariates. Significant connections were identified by calculating false discovery rate (FDR)-rate-corrected *p* < 0.05. Owing to the exploratory nature of this study, we used FDR correction for multiple comparison correction rather than Bonferroni correction (statistical significance, *p* < 0.0033) based on the number of ROIs in the SN, DMN, and FPN. It is because we considered it more appropriate to prioritize statistical power over strict control of Type I error. When applying FDR correction, a single FDR correction was applied to all comparisons across the three networks (SN-DMN, SN-FPN, and FPN-DMN).

#### 2.6.2 Inter-network FC analyses

We assessed the associations between the GPIUS-2 total score and its subscales and the FC values between the SN and DMN, SN and FPN, and FPN and DMN. The Kolmogorov-Smirnov tests were performed to test whether the inter-network FC strengths were normally distributed. Furthermore, a partial correlation analysis was performed by controlling for age, sex, and BDI-II using SPSS Version 22.0.0.

## 3 Results

### 3.1 Demographic information and psychological data

Three participants were excluded because of head motion, and 116 were included in the psychological data analysis. The demographic data are summarized in Table 1. The mean GPIUS-2 score was 36.78 (*SD* = 15.91), which was lower than the mean score (GPIUS-2 = 40.53) from a previous PIU study (Yoshitake, 2018). The mean values were below the BDI-II cut-off points. The Kolmogorov-Smirnov tests revealed that age, POSI, MOOD, SELF, NEGA, and BDI-II were not normally distributed. Therefore, Spearman’s ρ was used to analyze the correlation between each GPIUS-2 score and BDI-II.

**TABLE 1:** Demographic data.

	*N* = 116
Men (%)	73 (62.9%)
Mean age	35.78 ± 14.20
Mean GPIUS-2	36.78 ± 15.91
Mean POSI	5.76 ± 2.64
Mean MOOD	5.76 ± 5.00
Mean SELF	15.37 ± 7.27
Mean NEGA	6.64 ± 3.71
Mean BDI-II	6.33 ± 5.96

GPIUS-2, Generalized Problematic Internet Use-2; POSI, preference for online social interaction; MOOD, mood regulation; SELF, self-regulation; NEGA, negative outcome; BDI-II, Beck Depression Inventory-II.

**FIGURE 1:**
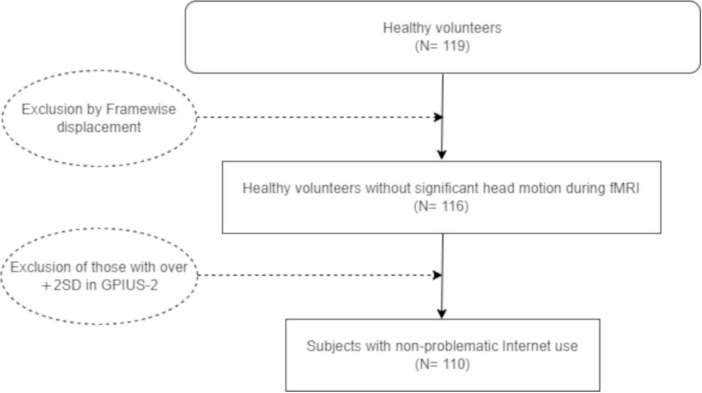
Flow chart of recruitment and exclusion. SD, standard distribution; GPIUS-2, General Problematic Internet Use Scale-2; fMRI, functional magnetic resonance imaging.

Correlation analysis revealed that neither the GPIUS-2 total scores nor each subscale, except for the SELF, correlated with the BDI-II scores. However, the SELF subscale scores were positively correlated with the BDI-II scores (Table 2). The student’s *t*-test revealed no difference in the total GPIUS-2 scores between sexes [*t*(114) = 0.975, *p* = 0.331]. The Mann–Whitney U test also revealed no differences in age and each subscale between the sexes (age, *p* = 0.527; POSI, *p* = 0.132; MOOD, *p* = 0.567; SELF, *p* = 0.114; NEGA, *p* = 0.153; and BDI-II score, *p* = 0.945). Since the GPIUS-2 scale has no cut-off point, we excluded patients with GPIUS-2 scores above +2*SD* (total GPIUS-2 score, 68.60 points). The remaining 110 participants were classified as non-PIUs. The participant recruitment flowchart is summarized in Figure 1.

**TABLE 2:** Correlation between GPIUS-2 and BDI-II.

	BDI-II	
	Spearman’s ρ	*p*-value
GPIUS-2 total score	0.176	0.059
POSI	0.083	0.376
MOOD	0.085	0.362
SELF	0.236*	0.011*
NEGA	0.110	0.239

GPIUS-2, Generalized Problematic Internet Use-2; POSI, preference for online social interaction; MOOD, mood regulation; SELF, self-regulation; NEGA, negative outcome; BDI-II, Beck Depression Inventory-II.

*Statistical *p* < 0.05 was considered statistically significant.

### 3.2 Correlation between the GPIUS-2 score and FC

#### 3.2.1 ROI-to-ROI FC analyses

**FIGURE 2:**
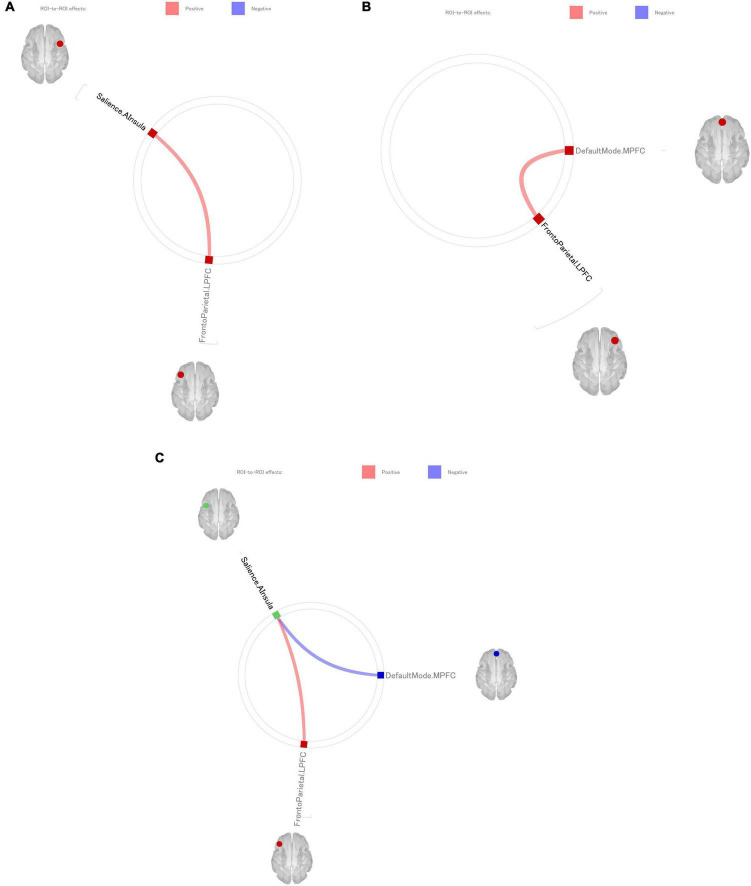
Functional connectivity (FC) with a significant correlation between “MOOD” subscale scores of the GPIUS-2 and resting-state connectivity (FDR-corrected, *p* < 0.05). The red line indicates a positive correlation between FC during the resting state and “MOOD” scores. Conversely, the blue line indicates a negative correlation between FC during the resting state and “MOOD” scores. **(a)** The “MOOD” score of the GPIUS-2 showed a significantly positive correlation with FC between the left anterior insula and the left LPFC. Conversely, the “MOOD” score showed a significantly negative correlation with FC between the left anterior insula and the MPFC. **(b)** The “MOOD” score showed a significant positive correlation with FC between the right anterior insula and left LPFC [*T*_(105)_ = 3.27, *p* = 0.0204]. **(c)** The “MOOD” score showed a significantly positive correlation with FC between the MPFC and right LPFC. MOOD, GPIUS-2 subscale of using the Internet for mood regulation; GPIUS-2, General Problematic Internet Use Scale-2; FDR, false discovery rate; ROI, region of interest; MPFC, medial prefrontal cortex; LPFC, lateral prefrontal cortex.

The correlations between the GPIUS-2 scores and each ROI in the three networks are shown in Figure 2. CONN toolbox analysis revealed the following: (1) the MOOD score of the GPIUS-2 showed a significantly positive correlation with FC between the left anterior insula (SN) and left LPFC (FPN) [*T*_(105)_ = 3.46; *r* = 0.172, *p* = 0.0109] (Figure 2a); (2) the MOOD score showed a significantly negative correlation with FC between the left anterior insula and MPFC (DMN) [*T*_(105)_ = −2.87, *r* = −0.159, *p* = 0.0351] (Figure 2a); (3) the MOOD score showed a significantly positive correlation with FC between the right anterior (SN) insula and left LPFC [*T*_(105)_ = 3.27, *r* = 0.168, *p* = 0.0204] (Figure 2b); and (4) the MOOD score showed a significant positive correlation with FC between the MPFC and right LPFC (FPN) [*T*_(105)_ = 3.42, *r* = 0.171, *p* = 0.0125] (Figure 2c). However, there were no significant associations between MOOD scores and intra-network FC values in the DMN, FPN, and SN.

#### 3.2.2 Inter-network FC analyses

The results of inter-network FC analyses are summarized in Table 3. The Kolmogorov-Smirnov tests revealed that each inter-network FC were normally distributed. Therefore, a partial correlation analyses was performed using Pearson’s by controlling for age, sex, and BDI-II. There was no significant correlation between the GPIUS-2 scores and internetwork FC among the three large-scale networks.

**TABLE 3:** Correlation between each inter-network integrity and GPIUS-2.

	SN-DMN	SN-FPN	DMN-FPN
GPIUS-2 total score (correlation efficient/*p*-value)	0.037 0.705	0.085 0.387	0.084 0.392
POSI (correlation efficient/*p*-value)	0.010 0.919	0.009 0.927	0.026 0.791
MOOD (correlation efficient/*p*-value)	0.059 0.549	0.046 0.639	0.156 0.109
SELF (correlation efficient/*p*-value)	0.039 0.691	0.107 0.276	0.037 0.710
NEGA (correlation efficient/*p*-value)	−0.064 0.514	0.018 0.853	0.060 0.538

GPIUS-2, Generalized Problematic Internet Use-2; POSI, preference for online social interaction; MOOD, mood regulation; SELF, self-regulation; NEGA, negative outcome; SN, salience network; DMN, default mode network; FPN, frontoparietal network. A *p*-value < 0.05 was considered statistically significant.

In summary, statistically significant correlations between Internet Use and FCs were found in ROI-to-ROI FC analyses, i.e., FCs between ROIs that are involved in the DMN, SN, and FPN were associated with the tendency of Internet use in ROI-to-ROI analysis.

## 4 Discussion

We analyzed the correlation between Internet use assessed using the GPIUS-2 and FC of inter- and intra-networks of the SN, DMN, and FPN using rsfMRI in non-PIU individuals. The mean GPIUS-2 total score was lower than that previously reported in a study of PIU, and the mean BDI-II score was lower than the cutoff point. It was confirmed that the participants were subclinical Internet users and were not depressed at the clinical level, in contrast to the fact that people with PIU have a high rate of comorbid depression. SELF scores were positively correlated with BDI-II scores, suggesting that internet use with higher but less than moderate levels of self-dysregulation was utilized as a coping strategy, as neither SELF nor depression levels reached a clinical level in this study sample. The results of ROI-to-ROI FC analyses revealed that the MOOD score showed (1) a significantly positive correlation with FC between the left anterior insula (SN) and left LPFC (FPN); (2) a significantly negative correlation with FC between the left anterior insula (SN) and MPFC (DMN); (3) a significantly positive correlation with FC between the right anterior insula (SN) and left LPFC (FPN); and (4) and significantly positive correlation with FC between the MPFC (DMN) and right LPFC (FPN). However, no significant relationship was found between the tendency toward Internet use and FCs in each network, contrary to our hypothesis.

Despite the negative results of the internetwork FC analyses, those of the ROI-to-ROI FC analyses can be discussed in the context of a triple-network model. Results (1)–(3) are inconsistent with those of most previous studies on PIU: increased FC between the SN and DMN and decreased FC between the SN and FPN in problematic Internet users compared with healthy controls (Chen et al., 2016; Hong et al., 2018; Yuan et al., 2016; Zhang et al., 2017). Results (1)–(3) were also inconsistent with a report on gambling disorder (Tsurumi et al., 2020). In contrast, these results are consistent with previous reports of elevated FC between the SN and FPN in expert video gamers (Caplan, 2010). Internet use is generally discussed in the context of behavioral addiction, and PIU has the same alterations in the internetwork integrity of the triple network as those in other psychiatric disorders, such as depression and schizophrenia (Manoliu et al., 2013; Shao et al., 2018). However, our results allow us to speculate that non-PIU is associated with an opposite modulation of psychiatric disorders in the internetwork FC of the triple network, which is responsible for affect and cognition and that non-PIU is a health-promoting habit. There are reports that cognitive load caused by non-PIU has a promoting effect on various brain functions. Internet users show increased activity in brain regions associated with decision-making and complex reasoning during internet use compared to non-users, and this activity further improves after training using internet search (Small et al., 2020). Additionally, computer-based brain training exercises have been reported to improve delayed memory and working memory abilities (Miller et al. 2013; Halford et al., 2007). Furthermore, groups that underwent multitasking training through video games showed improved abilities in working memory, divided attention, and sustained attention compared to those who did not (Anguera et al., 2013). Brain imaging studies have reported that the tendency of media multitasking, based on the Internet environment in many cases, was associated with higher attention network integrity (Kobayashi et al., 2020). The results of our current study support previous reports that appropriate level of internet use including computer-based brain training exercises improve working memory, attention functions, and multitasking abilities compared to groups that do not use them, from the perspective of FC.

Regarding the FC between the SN and FPN, an increase in FC has been reported to be associated with psychological benefits. In contrast, a decrease in FC has been reported in various psychiatric disorders, including substance dependence (Wilcox et al., 2019). Moreover, expert video gamers showed increased FC between the SN and FPN. This factor is potentially associated with higher cognitive functions including attention and working memory, which are required to play video games (Caplan, 2010). Furthermore, elevated FC between the SN and the FPN protects against cocaine relapse (McHugh et al., 2017). Regarding mood regulation, lower FC of the SN-FPN is associated with repetitive negative thinking in patients with remitted major depression (Lydon-Staley et al., 2019), presumably because it functions as a hub of cognitive control (Marek and Dosenbach, 2018), and there is an interaction between cognition and mood response, as shown in the Person-Affect-Cognition-Execution model (Brand et al., 2019). Not only the above interpretation, but FPN has also been reported to be involved in various cognitive processes such as attention control or working memory (Schimmelpfennig et al., 2023; Sridharan et al., 2008). Therefore, it will be necessary to examine specific functions using fMRI tasks related to each cognitive function in the future.

Similarly, when focusing on FPN functions in mood regulation, one possible interpretation is that non-problematic Internet users might have elevated SN-FPN FC due to habitual Internet use, presumably aiming to regulate mood, resulting in successful coping with daily stress through more effective emotion recognition and processing. However, the causality between Internet use and FC remains unclear.

Depression is a common comorbid disorder in PIU, as shown in previous meta-analyses (Bozkurt et al., 2013; Ko et al., 2008). However, our results might be interpreted as a primary care benefit of Internet use below the intermediate level, considering that most Internet users would utilize the Internet at non-problematic levels and can regulate their own mood as a coping strategy against stress throughout their social lives.

The FC between the DMN and FPN was elevated during a divergent thinking task and was associated with creative cognition (Beaty et al., 2015). Moreover, the DMN and FPN cooperate during mind wandering (Andrews-Hanna et al., 2014; Christoff et al., 2009), and sufficient mind wandering can promote high performance during technological use (Sullivan and Davis, 2020). From our results, it can be said that an increase in FC between the DMN and FPN contributes to beneficial mind wandering and may support cognitive performance. Additionally, elevated FC in patients with DMN-FPN has been reported to lower the risk of depression (Mu et al., 2024). Our study results showed that higher DMN-FPN FC in individuals with higher (but non-problematic) levels of Internet use could be explained at a higher level in creative thinking or mind wandering, even in resting-state conditions, although further studies are needed to compare PIU and non-PIU in this context.

In addition to the network, each ROI identified in this study has been reported to be associated with mood regulation. From the perspective of regional brain function, the results of the ROI-to-ROI FC analyses (section 3.2.1) in relation to the anterior insula, LPFC, and MPFC are consistent with those of previous studies regarding mood regulation. Evidence shows that the anterior insula is involved in mood regulation and plays an important role in controlling and allocating resources to the FPN. In addition, the bilateral insula is a key region of the SN that has been reported to be associated with mood. A meta-analysis of fMRI studies has revealed that the anterior insula is involved in the processing of cognitive and social emotions (Kurth et al., 2010). Moreover, this region is important for dealing with subjective emotional states (Namkung et al., 2017; Uddin, 2015). It has been reported that the mood-improving effects of mindfulness may be associated with increased blood flow in the insular cortex (Sezer et al., 2022; Tang et al., 2015). The LPFC and MPFC are involved in cognitive and emotional processing, and mood regulation (Smith et al., 2018; Tully et al., 2014). The left LPFC has been suggested to be a region of the circuit for emotion regulation (Grabell et al., 2019; Ochsner et al., 2012; Sarkheil et al., 2015). One possible interpretation is that the current study’s the bilateral insular and synchronized LPFC activity resulted in well-functioning emotional regulation as an adequate coping strategy for individuals without PIU.

This study had several limitations. First, the causal relationship between non-PIU and FC in the SN and FPN is still unknown because of the cross-sectional nature of this study. Second, we did not consider the purpose of internet use, such as social networking services and gaming. There is still debate about whether online games, gambling, and pornography fall under the category of PIU, because the target behavior itself is addictive. Whether such behaviors are an issue of online use should be clarified in the future by examining individual lifestyle behaviors online. Such information can improve Internet literacy. Third, the GPIUS-2 total scores were not correlated with FC, although a significant relationship was found between mood regulation subscale scores and FC. Therefore, we cannot conclude that subclinical internet use generally facilitates FC. Further studies are warranted to clarify the causal relationship between Internet use and brain function as a representation of mental health using a longitudinal design with a larger sample size for each online behavioral problem. Fourth, the relatively shorter scan time of 360 s for resting fMRI may lead to inadequate capture of low-frequency oscillatory signals (0.01–0.1 Hz) in particular. We believe that future studies should be conducted with longer imaging time. Fifth, the spatial resolution of the functional images obtained in this study not very high. This may cause partial volume effects in some brain regions. However, since the target areas in this study were representative core regions and did not target fine structures, the bias is considered to be relatively small. Sixth, it is necessary for more accurate correction of physiological noise. Although the CompCor used in this study has been shown to be able to reduce physiological noise such as cardiac respiratory related-noise (Behzadi et al., 2007), it is desirable to introduce physiological noise correction by simultaneous measurement of physiological data in our future study. On the other hands, the strength of our study is that it is the first to investigate the alterations in the FC of triple networks in non-PIU patients.

Our findings can be summarized as follows. First, the direction of the correlation of FC among the three core networks with the score of Internet use for mood regulation in non-PIU was opposite to the change in FCs in PIU. Second, most of the regions comprising the networks that showed a correlation with the Internet use score for mood regulation in non-PIU individuals were regions known to be involved in mood regulation. These results suggest that the resting-state FC of the SN-FPN and DMN-FPN can be used as potential biomarkers for distinguishing the safe use of the Internet from PIU. If the tendency to use the Internet for mood regulation is low to moderate, triple-network FC could be associated with better cognitive processing, potentially resulting in better mental health from the viewpoint of neural connectivity. We believe that these results will provide insight into Internet use, thus establishing ideal Internet literacy with safety and efficacy and appropriate risk assessment of PIU from the perspective of addictive problems. For example, considering FC alterations as a biomarker, and monitoring individuals at risk for PIU for MOOD scores in the appropriate range through periodic GPIUS-2 assessments may have a potential to contribute to mental health management. Furthermore, as reported in substance dependence, interventions involving rTMS targeting areas with impaired functional connectivity may be effective (Amiaz et al., 2009).

## Data Availability

The raw data supporting the conclusions of this article will be made available by the authors, without undue reservation.

## Footnotes

1
www.nitrc.org/projects/conn

2
https://www.fil.ion.ucl.ac.uk/spm

3
https://www.fmrib.ox.ac.uk/fsl

4
www.nitrc.org/projects/conn

5
https://www.fil.ion.ucl.ac.uk/spm

6
www.nitrc.org/projects/artifact_detect/
